# A Cauchy type inequality for Möbius operations

**DOI:** 10.1186/s13660-018-1690-2

**Published:** 2018-04-24

**Authors:** Keiichi Watanabe

**Affiliations:** Department of Mathematics, Faculty of Science, Niigata University, Niigata, Japan

**Keywords:** 26D15, 40A05, Cauchy inequality, Convergence of series, Möbius addition, Möbius scalar multiplication

## Abstract

In this article, we show two fundamental features of the restriction of Möbius operations to the real numbers, that is, a Cauchy type inequality and a criterion for convergence of series.

## Introduction and preliminaries

Möbius addition is defined on the complex open unit disk $\Bbb {D} = \{z \in\Bbb {C} ; |z| < 1\}$ by

$$ a \oplus b = \frac{a + b}{1 + \overline{a}b} \quad(a, b \in\Bbb {D}), $$

which appears in a wide variety of fields of mathematics. In particular, although Möbius addition is known in the literature as a *hyperbolic translation*, its group-like structure had gone unnoticed until it was uncovered by A.A. Ungar in 1988 [1], in the context of Einstein’s special theory of relativity. Furthermore, Ungar extended Möbius addition, introduced Möbius scalar multiplication to open balls of arbitrary real inner product spaces and established the concept of gyrovector spaces, which have a vector space-like structure (see [2–4]).

In this article, we show two fundamental features of the restriction of Möbius operations to the real numbers, that is, a Cauchy type inequality and a criterion for convergence of series.

### Definition 1.1

([5])

Let $s > 0$ be a fixed number. The addition $\oplus_{s}$ and scalar multiplication $\otimes_{s}$ on the open interval $(-s, s)$ in the real line are defined by the equations

$$\begin{gathered} a \oplus _{s} b = \frac{a + b}{1 + \frac{1}{s^{2}}ab}, \\ r \otimes _{s} a = s \tanh \biggl(r \tanh^{-1} \frac{a}{s} \biggr)\end{gathered} $$

for any $-s < a, b < s$, $r \in\Bbb {R}$. These operations appear in one of the axioms $(VV)$ of the real inner product gyrovector spaces defined by Ungar (see [2, Definition 6.2, Definition 6.83]).

Note that, on the interval $(-s, s)$, $\oplus_{s}$ is commutative, associative, and the operations $\oplus _{s}, \otimes_{s}$ together with the ordinary addition and multiplication have the following properties:

$$\begin{gathered} (r_{1}r_{2}) \otimes _{s} a = r_{1} \otimes _{s} (r_{2} \otimes _{s} a), \\ (r_{1} + r_{2}) \otimes _{s} a = r_{1} \otimes _{s} a \oplus _{s} r_{2} \otimes _{s} a, \\ r \otimes _{s} (a \oplus _{s} b) = r \otimes _{s} a \oplus _{s} r \otimes _{s} b\end{gathered} $$

for any $-s < a, b < s$, $r_{1}, r_{2}, r \in\Bbb {R}$.

### Proposition 1.2

(see [2, after Remark 3.41], [3, p. 1054])

*The Möbius addition* (*resp*. *Möbius scalar multiplication*) *reduces to the ordinary addition* (*resp*. *scalar multiplication*) *as* $s \to\infty$, *that is*,

$$\begin{gathered} a \oplus _{s} b \to a + b \quad(s \to\infty), \\ r \otimes _{s} a \to ra \quad(s \to\infty)\end{gathered} $$

*for any* $a, b, r \in\Bbb {R}$.

We simply denote ⊕_1_, ⊗_1_ by ⊕, ⊗, respectively. It is easy to see the following lemma, and we omit the proof.

### Lemma 1.3

([3, Theorem 4.2])

(i) *If* $0 < a, b < 1$, *then* $a \oplus b < a + b$.
(ii) *If* $-1 < a < a^{\prime}< 1$ *and* $0 < b < 1$, *then* $a \oplus b < a^{\prime} \oplus b$.

## Results and discussion

### A Cauchy type inequality

The classical Cauchy inequality for real numbers

$$ x_{1}y_{1} + \cdots+ x_{n}y_{n} \le \bigl(x_{1}^{2} + \cdots+ x_{n}^{2} \bigr)^{\frac{1}{2}} \bigl(y_{1}^{2} + \cdots+ y_{n}^{2} \bigr)^{\frac{1}{2}} $$

is one of the most fundamental inequalities in mathematics. In this section, we show an inequality of Cauchy type for Möbius operations.

Although some of the following lemmas might be well known, we present each proof for the sake of completeness and the convenience of readers.

#### Lemma 2.1

*If* *a*, *b* *are real numbers and* $c = (a^{2} + b^{2} )^{\frac{1}{2}}$, *then we have*

$$ \cosh c \le\cosh a \cdot\cosh b. $$

*The equality holds if and only if* $a = 0$ *or* $b = 0$.

#### Proof

It is straightforward to see

$$\begin{aligned} \cosh a\cdot\cosh b &= \biggl(1 + \frac{a^{2}}{2!} + \frac{a^{4}}{4!} + \cdots+ \frac{a^{2k}}{(2k)!} + \cdots \biggr)\\ &\quad{}\times \biggl(1 + \frac{b^{2}}{2!} + \frac{b^{4}}{4!} + \cdots+ \frac {b^{2k}}{(2k)!} + \cdots \biggr) \\ &= 1 + \frac{a^{2} + b^{2}}{2!} + \frac{a^{4} + 6a^{2}b^{2} + b^{4}}{4!} + \cdots\\ &\quad{} + \frac{a^{2k} + \cdots+ c_{j}a^{2(k -j)}b^{2j} + \cdots+ b^{2k}}{(2k)!} + \cdots,\end{aligned} $$

where the coefficient of the term $c_{j}a^{2(k -j)}b^{2j}$ in the numerator is

$$c_{j} = \frac{(2k)!}{(2k -2j)!(2j)!} \ge\frac{k!}{(k -j)!j!} = \binom{k}{j}. $$

So we obtain

$$\begin{aligned} \cosh a\cdot\cosh b &\ge1 + \frac{a^{2} + b^{2}}{2!} + \frac{a^{4} + 2a^{2}b^{2} + b^{4}}{4!} + \cdots\\ &\quad{} + \frac{a^{2k} + \cdots+ \binom{k}{j}a^{2(k -j)}b^{2j} + \cdots+ b^{2k}}{(2k)!} + \cdots \\ &= 1 + \frac{c^{2}}{2!} + \frac{c^{4}}{4!} + \cdots+ \frac {c^{2k}}{(2k)!} + \cdots \\ &= \cosh c.\end{aligned} $$

It is obvious that $a = 0$ or $b = 0$ implies the equality in the formula above. Conversely, if the equality holds, then we must have

$$\frac{a^{4} + 6a^{2}b^{2} + b^{4}}{4!} = \frac{a^{4} + 2a^{2}b^{2} + b^{4}}{4!}, $$

which implies that $a = 0$ or $b = 0$. This completes the proof. □

#### Lemma 2.2

*If* *a*, *b* *are real numbers and* $c = (a^{2} + b^{2} )^{\frac{1}{2}}$, *then we have*

$$ (\tanh c )^{2} \le (\tanh a )^{2} \oplus (\tanh b )^{2}. $$

*The equality holds if and only if* $a = 0$ *or* $b = 0$.

#### Proof

We have to show

$$ \biggl(\frac{e^{c} -e^{-c}}{e^{c} + e^{-c}} \biggr)^{2} \le\frac{ (\frac{e^{a} -e^{-a}}{e^{a} + e^{-a}} )^{2} + (\frac{e^{b} -e^{-b}}{e^{b} + e^{-b}} )^{2}}{ 1 + (\frac{e^{a} -e^{-a}}{e^{a} + e^{-a}} )^{2} (\frac {e^{b} -e^{-b}}{e^{b} + e^{-b}} )^{2}}, $$

which is equivalent to

1 $$\begin{aligned}[b] \frac{e^{2c} -2 + e^{-2c}}{e^{2c} + 2 + e^{-2c}} &\le\frac{(e^{a} -e^{-a})^{2}(e^{b} + e^{-b})^{2} + (e^{b} -e^{-b})^{2}(e^{a} + e^{-a})^{2}}{ (e^{a} + e^{-a})^{2}(e^{b} + e^{-b})^{2} + (e^{a} -e^{-a})^{2}(e^{b} -e^{-b})^{2}} \\ &= \frac{(e^{2a} -2 + e^{-2a})(e^{2b} + 2 + e^{-2b}) + (e^{2b} -2 + e^{-2b})(e^{2a} + 2 + e^{-2a})}{ (e^{2a} + 2 + e^{-2a})(e^{2b} + 2 + e^{-2b}) + (e^{2a} -2 + e^{-2a})(e^{2b} -2 + e^{-2b})}. \end{aligned} $$

It is straightforward to see that the numerator and denominator of the right-hand side in the formula (1) are respectively

$$ 2\bigl(e^{2a + 2b} + e^{2a -2b} -4 + e^{-2a + 2b} + e^{-2a -2b}\bigr) $$

and

$$ 2\bigl(e^{2a + 2b} + e^{2a -2b} + 4 + e^{-2a + 2b} + e^{-2a -2b}\bigr). $$

Hence, to prove inequality (1), it is sufficient to show the following inequality:

$$ \frac{e^{2c} -2 + e^{-2c}}{e^{2c} + 2 + e^{-2c}} \le\frac{e^{2a + 2b} + e^{2a -2b} -4 + e^{-2a + 2b} + e^{-2a -2b}}{ e^{2a + 2b} + e^{2a -2b} + 4 + e^{-2a + 2b} + e^{-2a -2b}}, $$

which is equivalent to

2 $$\begin{aligned}[b] &\bigl(e^{2c} -2 + e^{-2c}\bigr) \bigl(e^{2a + 2b} + e^{2a -2b} + 4 + e^{-2a + 2b} + e^{-2a -2b}\bigr) \\ &\quad\le\bigl(e^{2c} + 2 + e^{-2c}\bigr) \bigl(e^{2a + 2b} + e^{2a -2b} -4 + e^{-2a + 2b} + e^{-2a -2b} \bigr).\end{aligned} $$

It is easy to see that (2) is equivalent to

$$ \frac{e^{2c} + e^{-2c}}{2} \le\frac{e^{2a} + e^{-2a}}{2} \cdot\frac{e^{2b} + e^{-2b}}{2}, $$

which actually holds by *Lemma* *2.1*, because if $c = (a^{2} + b^{2})^{\frac{1}{2}}$, then we have

$$\bigl\{ (2a)^{2} + (2b)^{2} \bigr\} ^{\frac{1}{2}} = \bigl(4a^{2} + 4b^{2}\bigr)^{\frac{1}{2}} = 2 \bigl(a^{2} + b^{2}\bigr)^{\frac{1}{2}} = 2c. $$

The equality condition also follows from the previous lemma. This completes the proof. □

#### Lemma 2.3

*If* $a_{1}, \ldots, a_{n}$ *are real numbers*, *then we have*

3 $$ \tanh \bigl\{ \bigl(a_{1}^{2} + \cdots+ a_{n}^{2} \bigr)^{\frac{1}{2}} \bigr\} \le \bigl\{ (\tanh a_{1} )^{2} \oplus \cdots \oplus (\tanh a_{n} )^{2} \bigr\} ^{\frac{1}{2}}. $$

*The equality holds if and only if* $a_{j} = 0$ *except at most one* *j*.

#### Proof

For $n = 2$, the lemma is just *Lemma* *2.2*. Suppose that the lemma is valid up to *n*. Put $a = (a_{1}^{2} + \cdots+ a_{n}^{2} )^{\frac{1}{2}}$. Then *Lemma* *2.2* implies that

4 $$ \tanh \bigl\{ \bigl(a^{2} + a_{n+1}^{2} \bigr)^{\frac{1}{2}} \bigr\} \le \bigl\{ (\tanh a )^{2} \oplus (\tanh a_{n+1} )^{2} \bigr\} ^{\frac{1}{2}}. $$

By the assumption of our induction, we also have

5 $$ \tanh a = \tanh \bigl\{ \bigl(a_{1}^{2} + \cdots+ a_{n}^{2} \bigr)^{\frac{1}{2}} \bigr\} \le \bigl\{ (\tanh a_{1} )^{2} \oplus \cdots \oplus (\tanh a_{n} )^{2} \bigr\} ^{\frac{1}{2}}. $$

As an immediate consequence of (4) and (5), we obtain (3) for $n + 1$.

The equality condition follows from *Lemma* *2.2* easily. This completes the proof. □

The following lemma is an immediate consequence of the additional formula of tanh, and we omit the proof.

#### Lemma 2.4

*If* $a_{1}, \ldots, a_{n}$ *are real numbers*, *then we have*

$$ \tanh a_{1} \oplus \cdots \oplus \tanh a_{n} = \tanh (a_{1} + \cdots+ a_{n} ). $$

For Möbius operations restricted to the open interval $(-1, 1)$, the following inequality is a counterpart to the celebrated Cauchy inequality.

#### Theorem 2.5

*If* $r_{1}, \ldots, r_{n} \ge0$ *and* $0 \le x_{1}, \ldots, x_{n} < 1$, *then we have*

6 $$ r_{1} \otimes x_{1} \oplus \cdots \oplus r_{n} \otimes x_{n} \le \bigl(r_{1}^{2} + \cdots+ r_{n}^{2} \bigr)^{\frac{1}{2}} \otimes \bigl(x_{1}^{2} \oplus \cdots \oplus x_{n}^{2} \bigr)^{\frac{1}{2}}. $$

*The equality holds if and only if one of the following conditions is satisfied*:

(i) $r_{j} = 0$ ($j = 1, \ldots, n$);
(ii) $x_{j} = 0$ ($j = 1, \ldots, n$);
(iii) $r_{j} = x_{j} = 0$ *except for precisely one* *j*.

#### Proof

Put $a_{j} = \tanh^{-1}x_{j}$ ($j = 1, \ldots, n$).

$$\begin{gathered} r_{1} \otimes x_{1} \oplus \cdots \oplus r_{n} \otimes x_{n} \\ \quad= \tanh\bigl(r_{1}\tanh^{-1}x_{1}\bigr) \oplus \cdots \oplus \tanh\bigl(r_{n}\tanh^{-1}x_{n}\bigr) \\ \quad= \tanh\bigl(r_{1}\tanh^{-1}x_{1} + \cdots+ r_{n}\tanh^{-1}x_{n}\bigr) \\ \quad\le\tanh \bigl\{ \bigl(r_{1}^{2} + \cdots+ r_{n}^{2} \bigr)^{\frac{1}{2}} \bigl(a_{1}^{2} + \cdots+ a_{n}^{2} \bigr)^{\frac{1}{2}} \bigr\} \\ \quad\le\tanh \bigl[ \bigl(r_{1}^{2} + \cdots+ r_{n}^{2} \bigr)^{\frac{1}{2}} \tanh^{-1} \bigl\{ \bigl(x_{1}^{2} \oplus \cdots \oplus x_{n}^{2} \bigr)^{\frac{1}{2}} \bigr\} \bigr] \\ \quad= \bigl(r_{1}^{2} + \cdots+ r_{n}^{2} \bigr)^{\frac{1}{2}} \otimes \bigl(x_{1}^{2} \oplus \cdots \oplus x_{n}^{2} \bigr)^{\frac{1}{2}},\end{gathered} $$

because of the definition of the operation ⊗, *Lemma* *2.4*, the ordinary Cauchy inequality, *Lemma* *2.3*, and the definition of ⊗ again.

Finally, it is obvious that the equality holds in (6) if one of the conditions (i)–(iii) is satisfied. Conversely, if the equality holds in (6), then, from the equality conditions of the ordinary Cauchy inequality and *Lemma* *2.3*, one has $(r_{1}, \ldots, r_{n})$ and $(a_{1}, \ldots, a_{n})$ are linearly dependent, and $a_{j} = 0$ except at most one *j*. It yields the condition stated above. This completes the proof. □

#### Remark 2.6

Let $s > 0$. It is immediate to see that

$$ \frac{a}{s} \oplus \frac{b}{s} = \frac{a \oplus _{s} b}{s} \quad\mbox{and} \quad r \otimes \frac{a}{s} = \frac{r \otimes _{s} a}{s} $$

for any $-s < a, b < s$, $r \in\Bbb {R}$.

If $r_{1}, \ldots, r_{n} \ge0$ and $x_{1}, \ldots, x_{n} \ge0$, then, for sufficiently large $s > 0$, we have

7 $$ r_{1} \otimes _{s} x_{1} \oplus _{s} \cdots \oplus _{s} r_{n} \otimes _{s} x_{n} \le \bigl(r_{1}^{2} + \cdots+ r_{n}^{2} \bigr)^{\frac{1}{2}} \otimes _{s} \bigl(x_{1}^{2} \oplus _{s^{2}} \cdots \oplus _{s^{2}} x_{n}^{2} \bigr)^{\frac{1}{2}}. $$

Moreover, by letting $s \to\infty$ in inequality (7), we can get the classical Cauchy inequality.

### A criterion for convergence of series

In this section, we show a criterion for convergence of series in Möbius addition.

#### Definition 2.7

$$\begin{gathered} T_{e}(2m -1) = \sum_{(0)}x_{j} + \sum_{(2)}x_{j_{1}}x_{j_{2}} + \cdots+ \sum_{(2l)}\prod_{k=1}^{2l}x_{j_{k}} + \cdots+ \sum_{(2m-2)}\prod _{k=1}^{2m-2}x_{j_{k}}, \\ T_{o}(2m -1) = \sum_{(1)}x_{j} + \sum_{(3)}x_{j_{1}}x_{j_{2}}x_{j_{3}} + \cdots+ \sum_{(2l+1)}\prod _{k=1}^{2l+1}x_{j_{k}} + \cdots+ \sum _{(2m-1)}\prod_{k=1}^{2m-1}x_{j_{k}}, \\ T_{e}(2m) = \sum_{(0)}x_{j} + \sum_{(2)}x_{j_{1}}x_{j_{2}} + \cdots+ \sum_{(2l)}\prod_{k=1}^{2l}x_{j_{k}} + \cdots+ \sum_{(2m)}\prod _{k=1}^{2m}x_{j_{k}}, \\ T_{o}(2m) = \sum_{(1)}x_{j} + \sum_{(3)}x_{j_{1}}x_{j_{2}}x_{j_{3}} + \cdots+ \sum_{(2l+1)}\prod _{k=1}^{2l+1}x_{j_{k}} + \cdots+ \sum _{(2m-1)}\prod_{k=1}^{2m-1}x_{j_{k}}.\end{gathered} $$

Here, each $\sum_{(q)}$ appearing in $T_{e}(p)$ or $T_{o}(p)$ indicates the sum of all combinations which choose *q* items from $x_{1}, \ldots, x_{p}$. Exceptionally, we define $\sum_{(0)}x_{j} = 1$. For example, $m = 2$

$$\begin{gathered} T_{e}(3) = \sum_{(0)}x_{j} + \sum_{(2)}x_{j_{1}}x_{j_{2}} = 1 + x_{1}x_{2} + x_{1}x_{3} + x_{2}x_{3}, \\ T_{o}(3) = \sum_{(1)}x_{j} + \sum_{(3)}x_{j_{1}}x_{j_{2}}x_{j_{3}} = x_{1} + x_{2} + x_{3} + x_{1}x_{2}x_{3}, \\ \begin{aligned}T_{e}(4) &= \sum_{(0)}x_{j} + \sum_{(2)}x_{j_{1}}x_{j_{2}} + \sum _{(4)}\prod_{k=1}^{4}x_{j_{k}} \\ &= 1 + x_{1}x_{2} + x_{1}x_{3} + x_{1}x_{4} + x_{2}x_{3} + x_{2}x_{4} + x_{3}x_{4} + x_{1}x_{2}x_{3}x_{4},\end{aligned} \\ \begin{aligned}T_{o}(4) &=\sum_{(1)}x_{j} + \sum_{(3)}x_{j_{1}}x_{j_{2}}x_{j_{3}} \\ &= x_{1} + x_{2} + x_{3} + x_{4} + x_{1}x_{2}x_{3} + x_{1}x_{2}x_{4} + x_{1}x_{3}x_{4} + x_{2}x_{3}x_{4}.\end{aligned}\end{gathered} $$

#### Lemma 2.8

*The following identities hold*:

8 $$\begin{aligned}& T_{o}(2m) + T_{e}(2m)x_{2m+1} = T_{o}(2m + 1), \end{aligned}$$

9 $$\begin{aligned}& T_{e}(2m) + T_{o}(2m)x_{2m+1} = T_{e}(2m + 1), \end{aligned}$$

10 $$\begin{aligned}& T_{o}(2m + 1) + T_{e}(2m + 1)x_{2m+2} = T_{o}(2m + 2), \end{aligned}$$

11 $$\begin{aligned}& T_{e}(2m + 1) + T_{o}(2m + 1)x_{2m+2} = T_{e}(2m + 2). \end{aligned}$$

#### Proof

For the first identity (8), it is immediate to see that

$$\begin{gathered} T_{o}(2m) + T_{e}(2m)x_{2m+1} \\ \quad= \Biggl\{ \sum_{(1)}x_{j} + \sum_{(3)}x_{j_{1}}x_{j_{2}}x_{j_{3}} + \cdots+ \sum_{(2l+1)}\prod _{k=1}^{2l+1}x_{j_{k}} + \cdots+ \sum _{(2m-1)}\prod_{k=1}^{2m-1}x_{j_{k}} \Biggr\} \\ \qquad{} + \Biggl\{ \sum_{(0)}x_{j} + \sum_{(2)}x_{j_{1}}x_{j_{2}} + \cdots+ \sum_{(2l)}\prod_{k=1}^{2l}x_{j_{k}} + \cdots+ \sum_{(2m)}\prod _{k=1}^{2m}x_{j_{k}} \Biggr\} x_{2m+1} \\ \quad= T_{o}(2m + 1).\end{gathered} $$

Other three identities (9), (10), and (11) can be obtained in the same manner. This completes the proof. □

#### Definition 2.9

Let $s > 0$ be a fixed number. For any sequence $\{x_{n}\}_{n}$ in the open interval $(-s, s)$, we say that a series

$$ x_{1} \oplus _{s} x_{2} \oplus _{s} \cdots \oplus _{s} x_{n} \oplus _{s} \cdots $$

converges if there exists $S \in(-s, s)$ such that $S_{n} \to S$, where the sequence $\{S_{n}\}_{n}$ is defined recursively by $S_{1} = x_{1}$ and $S_{n} = S_{n -1} \oplus _{s} x_{n}$. In this case, we say the series converges to *S* and denote

$$S = x_{1} \oplus _{s} x_{2} \oplus _{s} \cdots \oplus _{s} x_{n} \oplus _{s} \cdots. $$

#### Lemma 2.10

*If* $s = 1$, *then the equality* $\frac{T_{o}(n)}{T_{e}(n)} = S_{n}$ *holds for all* $n = 1, 2, \dots $.

#### Proof

Recall the definition $S_{n} = x_{1} \oplus \cdots \oplus x_{n}$. For $n = 1, 2$, we trivially have

$$\begin{gathered} S_{1} = x_{1} = \frac{T_{o}(1)}{T_{e}(1)}, \\ S_{2} = x_{1} \oplus x_{2} = \frac{x_{1} + x_{2}}{1 +x_{1}x_{2}} = \frac{T_{o}(2)}{T_{e}(2)}.\end{gathered} $$

Suppose that the lemma is valid up to $n \ge2$. Then, if $n = 2m$,

12 $$\begin{aligned}[b] S_{2m + 1} &= S_{2m} \oplus x_{{2m} + 1} = \frac{S_{2m} + x_{{2m}+1}}{1 + S_{2m}x_{{2m}+1}} = \frac{ \frac{T_{o}({2m})}{T_{e}({2m})} + x_{{2m}+1}}{ 1 + \frac {T_{o}({2m})}{T_{e}({2m})}x_{{2m}+1}} = \frac{ T_{o}({2m}) + T_{e}({2m})x_{{2m}+1}}{ T_{e}({2m}) + T_{o}({2m})x_{{2m}+1}} \\ &= \frac{T_{o}(2m + 1)}{T_{e}(2m + 1)}\end{aligned} $$

by identities (8) and (9). Moreover, if $n = 2m + 1$, then identity (12), which has just been established, identities (10) and (11) yield that

$$\begin{aligned} S_{2m+2} &= S_{2m+1} \oplus x_{2m+2} = \frac{S_{2m+1} + x_{2m+2}}{1 + S_{2m+1}x_{2m+2}} = \frac{ \frac{T_{o}(2m+1)}{T_{e}({2m+1})} + x_{2m+2}}{ 1 + \frac {T_{o}(2m+1)}{T_{e}(2m+1)}x_{2m+2}} \\ &= \frac{ T_{o}(2m+1) + T_{e}(2m+1)x_{2m+2}}{ T_{e}(2m+1) + T_{o}(2m+1)x_{2m+2}} \\ &= \frac{T_{o}(2m + 2)}{T_{e}(2m + 2)}.\end{aligned} $$

This completes the proof. □

The following theorem is a criterion for convergence of series in Möbius addition, which subsumes [6, Example 34].

#### Theorem 2.11

*Let* $\{x_{n}\}_{n=1}^{\infty}$ *be a sequence satisfying* $0 \le x_{n} < 1$ ($n = 1, 2, \ldots$). *Then the series* $x_{1} \oplus x_{2} \oplus \cdots \oplus x_{n} \oplus \cdots$ *converges in the open interval* $(-1, 1)$ *if and only if the series* $\sum_{n=1}^{\infty}x_{n} < \infty$ *in the ordinary sense*.

#### Proof

(⇒) Suppose that $x_{1} \oplus x_{2} \oplus \cdots \oplus x_{n} \oplus \cdots= S < 1$. It is trivial that $S_{n} \le S$ and that the sequence of ordinary partial sums $\{x_{1} + \cdots+ x_{n}\}$ is non-decreasing. Moreover, it follows from *Lemma* *2.10* that

$$\begin{aligned} x_{1} + \cdots+ x_{n} &\le\frac{x_{1} + \cdots+ x_{n}}{\prod_{j=1}^{n}(1 -x_{j})} \le \frac{T_{o}(n)}{\prod_{j=1}^{n}(1 -x_{j})} = \frac{T_{o}(n)}{T_{e}(n) -T_{o}(n)} = \frac{ \frac{T_{o}(n)}{T_{e}(n)}}{ 1 -\frac{T_{o}(n)}{T_{e}(n)}} \\ &= \frac{S_{n}}{1 -S_{n}} \le\frac{S}{1 -S}.\end{aligned} $$

Thus the sequence $\{x_{1} + \cdots+ x_{n}\}$ is bounded above, so that $\sum_{n=1}^{\infty}x_{n} < \infty$ in the ordinary sense.

(⇐) Suppose that $\sum_{n=1}^{\infty}x_{n} < \infty$. Then

$$\begin{aligned} d_{\mathrm{M}}(S_{n}, S_{m}) &= |S_{n} \oplus (-S_{m})| \\ &= (x_{1} \oplus \cdots \oplus x_{m} \oplus x_{m+1} \oplus \cdots \oplus x_{n}) \oplus \bigl\{ -(x_{1} \oplus \cdots \oplus x_{m})\bigr\} \\ &= x_{m+1} \oplus \cdots \oplus x_{n} \\ &\le x_{m+1} + \cdots+ x_{n} \to0 \quad(n > m \to \infty),\end{aligned} $$

by the definition of the function $d_{\mathrm{M}}$ (see [2, (6.288), p. 216]), the definition of the partial sum $S_{n}$ in the Möbius addition, commutativity and associativity of ⊕ on the interval $(-1, 1)$, $x_{n} \ge0$, and the assumption $\sum_{n=1}^{\infty}x_{n} < \infty$. Since the space $((-1, 1), d_{\mathrm{M}})$ is complete (see [6, Lemma 20 and Theorem 26]), there exists a value $0 \le S < 1$ such that $d_{\mathrm{M}}(S_{n}, S) \to 0$, which implies that $S = x_{1} \oplus x_{2} \oplus \cdots \oplus x_{n} \oplus \cdots$. This completes the proof. □

#### Remark 2.12

Let $\{x_{n}\}_{n=1}^{\infty}$ be a sequence satisfying $0 \le x_{n} < s$ ($n = 1, 2, \ldots$). Then the series $x_{1} \oplus _{s} x_{2} \oplus _{s} \cdots \oplus _{s} x_{n} \oplus _{s} \cdots $ converges in the open interval $(-s, s)$ if and only if the series $\sum_{n=1}^{\infty}x_{n} < \infty$ in the ordinary sense.

#### Corollary 2.13

*If* $0 \le x < 1$, *then the series* $x \oplus x^{2} \oplus \cdots \oplus x^{n} \oplus \cdots$ *converges in the interval* $(-1, 1)$.

## Conclusions

We proved two fundamental theorems, that is, a Cauchy type inequality and a criterion for convergence of series for the restriction of Möbius operations to the open interval. They have certain importance such as the classical Cauchy inequality and convergence of series have importance for the ordinary addition and multiplication in the real and complex numbers.
